# Diversity Effects on Productivity Are Stronger within than between Trophic Groups in the Arbuscular Mycorrhizal Symbiosis

**DOI:** 10.1371/journal.pone.0036950

**Published:** 2012-05-21

**Authors:** Alexander M. Koch, Pedro M. Antunes, John N. Klironomos

**Affiliations:** Department of Integrative Biology, University of Guelph, Guelph, Ontario, Canada; WSL Institute for Snow and Avalanche Research SLF, Switzerland

## Abstract

**Background:**

The diversity of plants and arbuscular mycorrhizal fungi (AMF) has been experimentally shown to alter plant and AMF productivity. However, little is known about how plant and AMF diversity interact to shape their respective productivity.

**Methodology/Principal Findings:**

We co-manipulated the diversity of both AMF and plant communities in two greenhouse studies to determine whether the productivity of each trophic group is mainly influenced by plant or AMF diversity, respectively, and whether there is any interaction between plant and fungal diversity. In both experiments we compared the productivity of three different plant species monocultures, or their respective 3-species mixtures. Similarly, in both studies these plant treatments were crossed with an AMF diversity gradient that ranged from zero (non-mycorrhizal controls) to a maximum of three and five taxonomically distinct AMF taxa, respectively. We found that within both trophic groups productivity was significantly influenced by taxon identity, and increased with taxon richness. These main effects of AMF and plant diversity on their respective productivities did not depend on each other, even though we detected significant individual taxon effects across trophic groups.

**Conclusions/Significance:**

Our results indicate that similar ecological processes regulate diversity-productivity relationships within trophic groups. However, productivity-diversity relationships are not necessarily correlated across interacting trophic levels, leading to asymmetries and possible biotic feedbacks. Thus, biotic interactions within and across trophic groups should be considered in predictive models of community assembly.

## Introduction

An important goal in community ecology is to understand the relationship between biodiversity and ecosystem functioning. Much of this research began with seminal studies that sparked a lasting research interest on the ecosystem-level consequences of local biodiversity [1], [2]. Many studies have focused on diversity-productivity relationships in plants, because they are the main primary producers at the base of food webs. Plants are also amenable to field and laboratory manipulations, and general results of such studies show that ecosystem productivity often asymptotically increases with plant diversity; but also see [3]. These positive relationships are typically explained by a sampling effect or by functional complementarity among coexisting species [4], [5]. In addition, functional and phylogenetic diversity have also been shown to affect ecosystem productivity [6], [7].

It is clear though that ecosystem productivity is not solely a function of plant community structure. Other trophic groups such as decomposers, pathogens, pollinators, herbivores and microbial symbionts may alter plant community structure and ecosystem functioning and contribute to productivity [8]–[12]. In the present study we focus on the interaction between plants and arbuscular mycorrhizal fungi (AMF), soil-dwelling symbionts that associate with the roots of most terrestrial plants [13]. The presence and diversity of AMF can influence plant diversity and productivity [14]–[16]. Mycorrhizal symbionts may receive significant amounts of photosynthates from their host plants, making them also an important component of the terrestrial carbon cycle [17]. Plant diversity was reported to influence AMF community structure and increase AMF abundance [18]–[20]. Conversely, plant diversity-productivity relationships were significantly altered by the presence of different AMF [21] or by AMF diversity [15], [16].

Thus far, manipulations of AMF and plant diversity have been done separately, which is why we have little understanding as to whether AMF and plant diversity are independently affecting community structure and ecosystem productivity. The main goal of the present study was to determine the effect of AMF and plant diversity, and their interaction on AMF and plant productivity. We experimentally co-manipulated initial plant and AMF community structure by establishing different plant monocultures and their respective plant mixtures in two complementary greenhouse experiments. We inoculated each host treatment by either morphologically distinct single AMF morphotypes or their mixture (Experiment 1), or by two different AMF mixtures, each comprised four genetically distinct AMF (either distinct or the same morphotypes), or both of these treatments combined (Experiment 2). At the end of the experiments we assessed both plant and AMF productivity by the total plant shoot biomass and the total extraradical fungal volume (EFV), respectively. We found positive diversity-productivity relationships within both trophic groups, without evidence for a significant interaction between AMF and plant diversity. The increase in AMF productivity in AMF mixtures was largely independent of plant productivity and went beyond what could be explained by a sampling effect. Different plant monocultures significantly altered EVF and AMF spore communities, whereas within plant mixtures AMF productivity appeared to be driven by the dominant plant species. Our results show that the interactions within and between trophic groups influence community structure and productivity of above- and belowground communities.

## Materials and Methods

### AMF and Plant Species Used

Morphotypes (isolates) of different AMF families were used to compare taxa of varying relatedness and growth strategies [7]. AMF were obtained from the International Culture Collection of Arbuscular Mycorrhizal Fungi (INVAM, http://invam.caf.wvu.edu/index.html) or axenic root organ cultures (ROC, see below). Prior to our experiments, INVAM cultures were grown for over a year with *Sorghum vulgare* (Pers.) var. sudanense under standardized conditions in a greenhouse at the University of Guelph (Canada). Pot cultures were assessed for presence of healthy-looking AMF spores, and absence of non-target AMF morphotypes was confirmed. INVAM isolates BR225 (*Acaulospora morrowiae* Spain & Schenk), NB114 (*Glomus mosseae* Nicolson & Gerd.), and SN722 (*Scutellospora heterogama* Nicolson & Gerd.) were used in Experiment 1, and UT183 (*Glomus etunicatum* Becker & Gerd.), UK126 (*G. mosseae*), WV858B (*S. heterogama*) and NC110A (*Gigaspora gigantea* Nicolson & Gerd.) in Experiment 2. Additionally, a volumetric 1∶1:1∶1 mixture of four closely related, but genetically distinct isolates (A4, B3, C2, and DAOM 197198) [22] was used in Experiment 2. Their culture history is described in detail in Koch *et al*. (2004). The inoculum was prepared by thoroughly mixing six 18-week old ROC plates (each containing 25 ml of M-medium, carrot host roots, AMF hyphae and spores) of each isolate. A recent genetic analysis re-classified these isolates as *G. irregulare* [23], a species that is morphologically and genetically closely related to *G. intraradices*.

The plant species *Daucus carota* L. (Apiaceae), *Prunella vulgaris* L. (Lamiaceae) and *Achillea millefolium* L. (Asteraceae) were used in Experiment 1. *A. millefolium*, *Bromus inermis* Leyss. (Poaceae), and *Medicago sativa* L. (Fabaceae) were used in Experiment 2. James Ferguson (Elora Research Station, University of Guelph, ON) provided seeds of *M. sativa*. The seeds from all other plant species were collected from a large number (>50) of randomly chosen individuals for each species at the Long-Term Mycorrhizal Research Site (LTMRS, located on the University of Guelph campus) in October 2006, air-dried, and pooled accordingly.

### Experimental Design and Set-up

Field soil was collected from one location at the LTMRS in October 2006, sieved (5 mm mesh width) and air-dried at room temperature. The substrate of Experiment 1 consisted of a 4∶3∶1 volumetric mixture of air-dried LTMRS soil, Turface (a montmorillonite clay, Turface Athletics MVP, Profile Products LLC, Buffalo Grove, IL, USA), and sand (Nepheline Syenite, Unimin Canada Ltd., Toronto, ON, Canada), and was pasteurized in an electric heating unit at 95°C for 1 hour. The substrate of Experiment 2 consisted of a volumetric 1∶3∶1 mixture of LTMRS soil, Turface, and washed Horticultural Sand (Hillview, Nu-Gro IP Inc., Brantford, ON, Canada), and was steam-autoclaved twice at 121°C for 45 minutes on separate days.

Experiment 1 comprised 120 pots (each filled with 1.4 L of substrate) with saucers in a completely randomized factorial design. The factor host community (three different monocultures and their mixture) was crossed with AMF inoculation, with six replicate pots for each possible combination. AMF inoculation treatments consisted of non-mycorrhizal controls, and inoculation by isolates belonging to *A. morrowiae*, *S. heterogama* and *G. mosseae*, or a mixture of these three isolates, respectively. For each of the three morphotypes, we extracted spores from 200 ml of substrate using sucrose gradient centrifugation. In all mycorrhizal treatments nine healthy AMF spores were added to each pot on 11 and 12 January 2007. Three similar-sized AMF spores were pipetted onto the germination root of each of three 1-week old seedlings of *S. vulgare* (pre-germinated on sterilized vermiculite), which were then transplanted. *S. vulgare*, the previous host of these isolates, was used as common “AMF starter host” to maximize AMF establishment. As a result, nine spores of either the same isolate (AMF monocultures) or three spores of each of the three morphotypes (AMF mixtures) were added to each AMF-inoculated pot. No AMF spores were added to *S. vulgare* seedlings of controls. The order of inoculation of individual pots was fully randomized. Pots were placed in random order on a greenhouse bench and re-randomized monthly. Each pot received 4 ml of a microbial filtrate to correct for potential differences in non-AMF microbial communities. The microbial filtrate consisted of an 800 ml H_2_O-slurry that included 10 g of substrate from each AMF culture, passed through a 20 µm mesh.

Seeds of the “target” host plants *D. carota*, *P. vulgaris,* and *A. millefolium* were surface-sterilized [24] and added to the pots on 13 January 2007. To keep the plant density identical in monocultures and mixtures, extra seedlings were removed on 8 February 2007 to leave three similar-sized and evenly spaced individual seedlings per pot. On 19 March 2007 all *S. vulgare* shoots were excised at the level of the substrate, dried (3 days, 70°C) and weighed. Subsequent analyses did not indicate that *S. vulgare* dry weight (dw) was significantly altered by any experimental factor (data not shown). Fertilizer (200 mg 17-5-19 Poinsettia) was added to each pot on both 4 April and 11 May 2007. The temperature in the greenhouse ranged from 18 to 30°C. Day length was 16 h, supplemented with artificial lights from 6 am to 10 pm when necessary. Pots were watered every 2–3 days to field capacity. On two consecutive days (1–2 May 2007), a cloudy and a sunny day, all pots were weighed after watering and reweighed 24 hours later. These measures were used to estimate whole-pot evapotranspiration rates to assess an additional eco-physiological measure other than plant and fungal productivity (see below). The experiment was harvested on 5–6 June 2007. Shoots were excised at the substrate level, dried and weighed. Individual plant species were only separated for plant mixtures. Root systems were washed and approximately 2 g (fresh weight) of root material was stored in 50% ethanol. Roots were stained and the percentage of root length colonized by AMF determined [25]. A sub-sample of substrate (≈100 ml) from each pot was air-dried and used for measures of extraradical hyphal length and spore counts for each AMF morphotype [26], [27].

Previous experiments with these *G. irregulare* isolates and other AMF species showed that host responses could differ among conspecific isolates [24], [28]–[30]. In Experiment 2, we used a mixture of 4 genetically different *G. irregulare* isolates (see above) to test whether a genetically diverse inoculum of this morphotype results in similar host responses as a mixture of unrelated of morphotypes. Since different *G. irregulare* isolates were shown to anastomose, exchange genetic information and recombine (see [31] and references therein) it is still unclear whether these fungal genotypes are part of a common mycelium or relatively distinct functional units. It was not our intention to assess the individual contribution of these isolates to productivity traits. Therefore, the reader should simply consider this morphotype as a genetically enriched isolate-mixture.

Unless specified otherwise, the design and methods used for Experiment 2 were as those stated above. The factor host was crossed with AMF inoculation (4 levels), with four replicate pots for each possible combination. AMF inoculation treatments were ± *G. irregulare* (either adding 15 ml of a mixture of four *G. irregulare* isolates, or the equivalent amount of AMF-free ROC medium), crossed with ± addition of a mixture of four AMF morphotypes of different AMF species (see above). Substrate (600 ml) from each of these four AMF morphotype cultures was mixed, and 60 ml of this inoculum was added to individual pots (or 60 ml of sterile substrate to controls, respectively). Seeds of the target hosts were added to the pots and covered by a thin layer of sterilized washed sand. The experiment started on 3 August 2007. Each pot received 30 mg of fertilizer on 12 September and 2 November 2007. The plant shoots were excised on 7 February 2008, three days after the last watering, and substrates were subsequently air-dried within pots for 6 weeks.

### Statistical Analyses

Data of the two experiments were analyzed separately using multivariate analysis of variance (MANOVA) of AMF and plant productivity traits followed by univariate ANOVA. To compare inoculated treatments with uninoculated controls, different AMF-inoculated treatments were pooled. However, we focused our analyses on comparisons of the different inoculated treatments, which omitted controls. Total plant shoot dry weight of hosts per pot, EFV (see below) and percent colonization of roots by AMF (Experiment 1 only) were used as surrogates of plant and AMF productivity. To estimate EFV we added the estimates of total AMF spore and hyphal volumes (SV, and HV, respectively). We approximated the SV of each morphotype by multiplying the spore abundance by the estimated spore volume for that species, assuming spherical spores and using the mean spore diameter values published on the INVAM web-page for each AMF species. HV was calculated from the measures of hyphal length density, assuming cylindrical hyphae (10 µm diameter) and a substrate density of 2 g cm^−3^.

Fixed models were used with the factors Host and AMF inoculation. In both experiments plant and AMF mixtures were considered a fourth level (along with the respective monocultures). To compare different monocultures in Experiment 1 AMF and plant mixtures were excluded. Since spores are both an AMF productivity and fitness trait [32] for each AMF morphotype we also assessed separate 2-way ANOVAs on spore abundance and Malthusian fitness (*i.e.,* spore abundance divided by the number of spores added to individual pots), with factors host, and inoculation by other AMF. Each of these analyses disregarded the AMF treatments where each respective morphotype was not added as inoculum. To assess the effects of AMF and plant diversity on AMF and plant productivity, different plant and AMF monocultures were pooled respectively, and a factorial model was analyzed using MANOVA and ANOVA where AMF diversity (AMF monocultures and mixtures) was crossed with the plant diversity (plant monocultures and mixtures). In addition, a repeated measures ANOVA was used to analyze evapotranspiration rates in Experiment 1. Analyses for Experiment 2 were similar, and AMF sporulation was assessed by ANOVA (*G. irregulare* spores) and MANOVA (spores of the four AMF morphotypes used as a mixture). When necessary, response variables were Box-Cox transformed to meet the test’s assumptions of normality and homoscedasticity. P-value protected contrast analyses (CA) were calculated to compare levels of specific interest. Because each of the many different analyses addressed specific questions, we focused the results section on significant treatment effects that are clearly reflected in the Figures. All statistical analyses were performed using the software JMP 5.0 (SAS Institute Inc. Cary, NC, USA).

## Results

### Experiment 1

#### Effects of AMF inoculation on productivity

**Figure 1:**
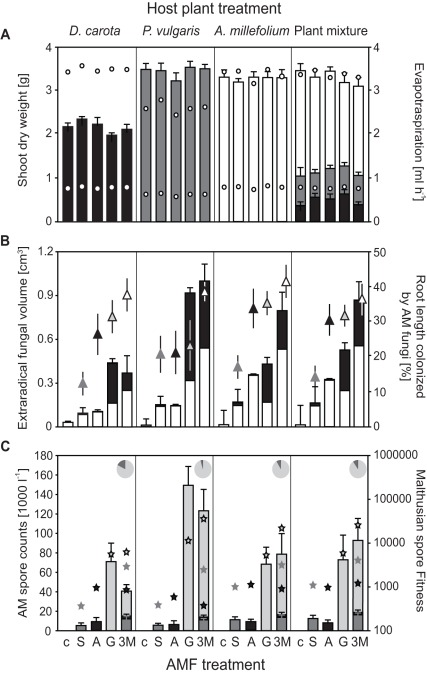
Effects of AMF inoculation and host plant treatments in Experiment 1. a) Total shoot dry weight (bars) and whole-pot evapotranspiration rates on a sunny and cloudy day (upper and lower open circles; the pooled SEMs were 0.0913 and 0.0192, respectively, and are not shown since they were smaller or about the same size than the symbols), b) total extraradical fungal volume per pot (bars; white shaded parts show the proportion of extraradical hyphal volume, the black shaded part shows the total AMF spore volume) and percent root length colonized by AMF (triangles), and c) AMF spore abundance (bars: number of AMF spores per 1 liter pot; pie charts indicate the relative abundance as total spore volume of the three morphotypes) and Malthusian fitness for each AMF isolate (stars, *i.e.* final AMF spore abundance divided by the number of spores added per pot as inoculum at the start). Error bars are SEM. Plant treatments are separated in sub-panels, and bars in a) are shaded for visual aid (black: *D. carota*; grey: *P. vulgaris*, white: *A. millefolium* in either monoculture or mixtures, where bars are stacked). Shades of bars in c) and symbols (b and c) are a visual aid to represent different AMF treatments (non-mycorrhizal controls [c], dark grey: *S. heterogama* [S], black: *A. morrowiae* [A], light grey *G. mosseae* [G]; white: AMF mixtures [3 M]).

**Figure 2:**
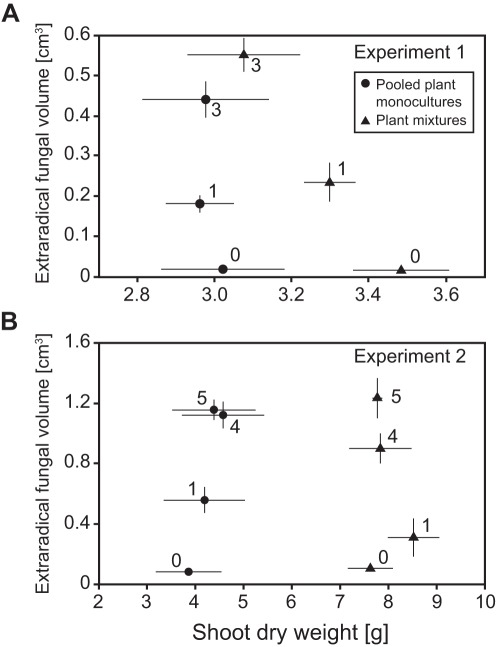
Relationships between productivity and diversity of AMF and host plants. Plant and fungal productivity measures (total mean shoot dry weight and total extraradical fungal volume, respectively) for plant and AMF diversity treatments in a) Experiment 1, and b) Experiment 2. Symbols show the overall means and SE of the pooled plant monocultures (dots) and mixtures (triangles) in the different AMF diversity treatments, for which numbers indicate the number of different AMF morphotypes used as inoculum: controls (0), only one AMF morphotype (1; in Experiment 1 the three different AMF monocultures were pooled), and AMF mixtures (3,4, or 5 AMF morphotypes were used).

Overall, the productivity of control plants did not significantly differ from that of AMF-inoculated plants (Fig. 1a and 2a). AMF inoculation, however, had a strong effect on fungal productivity. No AMF-specific structures were observed in controls, which also had much lower EFV than any of the inoculated treatments (Fig. 1, Fig. 2a). When analyzing only plant and AMF monocultures, productivity traits of both trophic groups combined (MANOVA on shoot dw, AMF root colonization, and EFV) were significantly altered by the factors host species (*F*_6,86_ = 12.86, *P*<0.0001), morphotype (*F*_6,86_ = 7.12, *P*<0.0001), and their respective interaction (*F*_12,132_ = 2.70, *P*  = 0.0027) (Fig. 1a and b). Univariate ANOVA confirmed these results, and significant host species and AMF morphotype by host species interaction effects (*F*_2,44_ = 132.62, *P*<0.0001 and *F*_4,44_ = 3.16, *P* = 0.0236, respectively) were also observed for plant productivity. These patterns were mainly the result of *G. mosseae* promoting less growth of *D. carota* than other AMF isolates. *G. mosseae* was also the most productive morphotype on all host species, both in terms of EFV and AMF root colonization levels. Overall *D. carota* promoted the least EFV, but AMF productivity depended on both morphotype and plant species identity, *i.e.* we detected significant host species (*F*_2,44_ = 3.61, *P* = 0.0353) and morphotype (*F*_2,44_ = 24.45, *P*<0.0001) effects, and a significant host species by morphotype interaction (*F*_4,44_ = 3.94, *P* = 0.0081).

When both AMF and plant mixtures were included, each of the three AMF monocultures had significantly lower ERV and root colonization rates than the AMF mixtures (CAs, for *G. mosseae*: *F*_1,78_ = 7.26, *P* = 0.0086 and *F*_1,78_ = 4.77, *P* = 0.0320, respectively, in all other cases *F*_1,78_≤10.14 and *P*≤0.0021). In contrast, AMF monocultures did not promote different plant growth than AMF mixtures. When comparing each plant monoculture to the plant mixtures, only *D. carota* had a reduced ERV (CA, *F*_1,78_ = 13.16, *P* = 0.0005). Similarly, aboveground plant productivity of *D. carota* monocultures was significantly decreased (CA, *F*_1,78_ = 227.91, *P*<0.0001), and that of *P. vulgaris* monocultures significantly increased (CA, *F*_1,78_ = 4.61, *P* = 0.0349), relative to the plant mixtures. To test effects of manipulated AMF and plant species diversity on fungal and plant productivity, AMF and plant monocultures were pooled (Fig. 2a). Plant productivity increased with plant diversity, but was not altered by AMF diversity. Conversely, EFV was strongly enhanced by AMF diversity (*F*_1,90_ = 32.69, *P*, 0.0001), but not by plant diversity. We found no significant interaction between AMF and plant diversity in MANOVA and univariate ANOVAs on these productivity measures.

### Effects on Evapotranspiration

The two measures of evapotranspiration were strongly correlated (*R*_Pearson_ = 0.9026), being overall 4.3 times higher on the sunny day (3.319 ml h^−1^ ± 0.038 SE, Fig. 1a). Plant treatments differed in evapotranspiration (*F*_3,80_ = 105.02, *P*<0.0001), with *P. vulgaris* having the lowest rates. AMF significantly affected evapotranspiration rates (*F*_3,80_ = 4.59, *P* = 0.0051) with plants inoculated by *A. morrowiae* and *S. heterogama* having overall the lowest and highest evapotranspiration rates, respectively. Evapotranspiration rates of AMF mixtures were intermediate, and controls did not significantly differ from AMF inoculated treatments.

#### Effects on AMF spore production

Spore size, abundance, and total spore volume differed among the tested AMF morphotypes (Fig. 1b and c). *A. morrowiae* produced the fewest and smallest spores. Its spore production was negatively affected by the presence of other morphotypes (*F*_1,39_ = 19.14, *P*<0.0001), while its Malthusian fitness was not significantly altered by any experimental factor. In contrast, *S. heterogama* almost doubled spore formation when growing in AMF mixtures compared to its monocultures (*F*_1,40_ = 14.28, *P* = 0.0005, Fig. 1c). *S. heterogama* spore counts were also altered by host treatment (*F*_3,40_ = 5.62, *P* = 0.0026) and were the highest when *A. millefolium* was present in the host community. *G. mosseae* was the AMF morphotype that produced the most spores, and its spores dominated in AMF mixtures (Fig. 1c). *G. mosseae* produced the most spores on *P. vulgaris* (*F*_3,39_ = 4.91, *P* = 0.0055), but was not significantly affected by the presence of other AMF morphotypes. Despite an increase in Malthusian fitness, overall *G. mosseae* spore production was reduced by almost 25% in AMF mixtures compared to its monocultures, and this effect was most pronounced in *D. carota* monocultures. No significant AMF x host treatment interaction was detected for any of these morphotypes.

### Experiment 2

#### Effects on plant and fungal productivity

**Figure 3:**
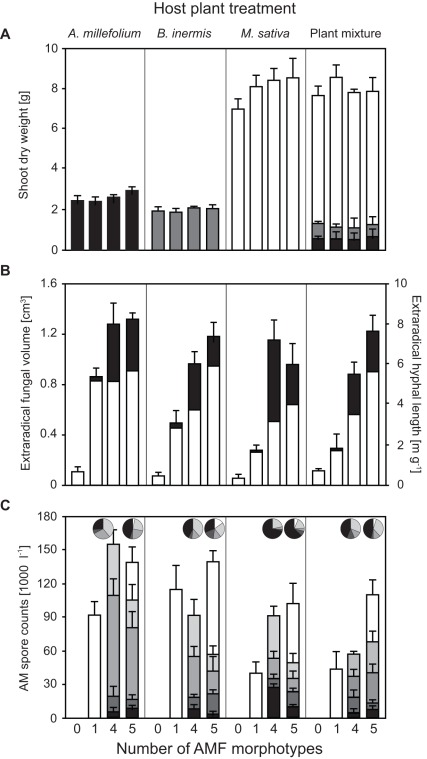
Effects of AMF inoculation and host plant treatments in Experiment 2. a) Total shoot dry weight, b) total estimated extraradical fungal volume (white: proportion of extraradical hyphal volume, black: total AMF spore volume), and c) spore abundance (number of AMF spores) of the different AMF morphotypes; for morphotypes-mixtures bars were stacked by phylogenetic distance from *G. irregulare* (white): *G. mosseae* (light grey), *G. etunicatum* (grey), *S. heterogama* (dark grey), and *G. gigantea* (black). The total dry weights of the three plants species monocultures are differently shaded in a). Pie charts in c) indicate relative total spore volume of morphotype mixtures. Error bars are SEM.

Plant productivity was mainly determined by host treatment (*F*_3,56_ = 492.77, *P*<0.0001) and inoculation by AMF (Fig. 3a), which increased plant productivity, especially of *M. sativa*, relative to non-inoculated controls (*F*_1,56_ = 6.62, *P* = 0.0127); the different AMF inoculation treatments did not statistically differ. *M. sativa* was the most productive plant monoculture, and dominated the plant mixtures. Plant mixtures were significantly more productive than the monocultures of *A. millefolium* and *B. inermis* (CAs, *F*_1,48_ = 913.62 and *F*_1,48_ = 1208.81, *P*<0.0001, respectively), but did not significantly differ from *M. sativa* monocultures.

As in Experiment 1, no AMF spores were detected in controls, which also had the lowest EFVs observed (Fig. 3b and c). Productivity traits of inoculated plant monocultures (MANOVA on plant dw and EFV) were altered by inoculation type (*F*_4,54_ = 9.03, *P*<0.0001) and different host species monocultures (*F*_4,72_ = 24.01, *P*<0.0001), but these factors did not significantly interact. The results were similar when plant mixtures were included. The plant productivity of both *B. inermis* and *A. millefolium* were less than that of *M. sativa* monocultures and plant mixtures, which were more productive than the pooled plant monocultures, irrespective of AMF inoculation (Fig. 2b and 3a). Fungal productivity (ANOVA on EFV) of AMF-inoculated pots was altered by host treatments (*F*_3,36_ = 6.49, *P*<0.0013) and was the highest on *A. millefolium* and *B. inermis* monocultures (Fig. 3b), plant species that have more finely branched root systems than *M. sativa* (unpublished data of the authors). Increasing plant species richness slightly reduced ERV, and AMF-productivity within plant mixtures appeared to be driven by the dominant *M. sativa*, which promoted the least AMF productivity as monocultures (Fig. 2b and 3b). However, as in Experiment 1, EFV was strongly driven by AMF morphotype richness (*F*_2,42_ = 28.52, *P*<0.0001). Of all inoculation treatments, *G. irregulare* monocultures promoted the least fungal productivity, yet addition of *G. irregulare* to the 4-morphotype mixture increased EFV in all host treatments but *M. sativa* monocultures. Other than AMF and plant diversity main effects, we detected no significant interaction on either plant or AMF productivity.

### Effects on AMF Spore Production

The spore production of *G. irregulare* was altered by host treatments (*F*_3,24_ = 4.52, *P* = 0.0119, Fig. 3c), but not by the presence of other AMF. Similarly, AMF spore abundances of the 4-morphotype mixture were also affected by host treatments (MANOVA, *F*_12,69_ = 2.73, *P* = 0.0044), but not by inoculation with *G. irregulare*. There was a trend, however, that AMF more closely related to *G. irregulare* (*i.e., G. mosseae* and *G. etunicatum*) were more negatively affected by its presence than the more distantly related isolates (*G. gigantea* and *S. heterogama*). Overall, total spore volumes of 4- and 5 morphotype mixtures were strongly correlated to the spore production of *G. gigantea* (*R_Pearson_* = 0.8025, *P*<0.0001), even though this morphotype was subordinate with regard to spore numbers (Fig. 3b and c).

## Discussion

Our study shows that AMF productivity was influenced by host community composition, supporting previous reports of significant host plant effects on AMF abundance [18], [20]. In our experiments, AMF productivity consistently increased with AMF richness, but was not influenced by plant productivity or plant species richness. Since the productivity of AMF mixtures was overall greater than of that of its individually measured constituents, the observed positive relationship between AMF diversity and productivity may not only be explained by a sampling effect [33], complementing recent findings of AMF diversity effects on plant productivity [34], [35]. Our results also show that different plant species affected individual AMF abundances (spore production), corroborating that host plants can induce significant AMF community changes [19], [36], [37].

In light of rapid global environmental change, there is an urgent need to improve our understanding of the fundamental processes that determine the abundance and distribution of organisms and the functioning of ecosystems. Many studies indicate that changes in climate, the spread of invasive species, or changes in land-use can have profound effects on ecosystem function. However, no single study can quantify all possible processes that impact individual species. For plant communities, some mechanisms, especially those involving belowground interactions, often remain unquantified. This is problematic, especially in light of recent data indicating that soil biota feedbacks may be more important plant community determinants than previously thought [38]–[40].

Regarding AMF, single and multi-isolate effects on plant growth are relatively well documented [15], [16], [24], [28]–[30], [34], [35], [41], [42]. Much less, however, is known about how plant species and their assemblages influence AMF productivity. Our results corroborate that both host community type and AMF diversity jointly drive AMF productivity [16], [18], [21], [42]. Even though individual AMF morphotypes did indeed have altered growth and symbiotic function when associating with different plant monocultures, we detected no significant interaction between AMF and host diversity on AMF and plant productivity. Positive diversity-productivity relationships typically arise from sampling effects or through functional niche complementarity. In the latter case, synergistic productivity effects are caused by functional dissimilarities among species due to a more efficient capture of available resources. An important novelty of our study is that we manipulated the diversities of two interacting trophic groups. The results of our two experiments provide evidence of both processes, especially in regards to AMF productivity. In Experiment 1, the most productive monoculture (*G. mosseae*) also dominated the AMF mixtures, but these mixtures were overall more productive than *G. mosseae,* particularly in terms of root colonization rates and extraradical hyphal production. Since host roots and soil are the main carbon and mineral nutrient sources for AM fungi, respectively, increasing AMF diversity resulted in a denser colonization of intra- and extraradical habitats by AMF, *i.e.* a seemingly more efficient resource capture.

Two additional observations drew our attention in Experiment 1. *S. heterogama* increased spore production in AMF mixtures relative to its monocultures, suggesting that interactions among AMF are not necessarily competitive and may include facilitation. Secondly, of all AMF monocultures, *A. morrowiae* produced the highest amounts of extraradical hyphae, but only in symbiosis with *A. millefolium*. Since the growth traits of these clonal fungi are also fitness traits [22], [32], these findings support that the success of different individual AMF structures depends on the biotic environment.

In a similar set-up as in Experiment 2, inoculation of unsterilized field soil by *G. irregulare* from ROCs decreased the diversity of native AMF inside host roots [28]. This suppressive effect may have been due to the strong inoculum potential of ROCs at the onset of the experiment. In our Experiment 2 no similar suppressive effect was observed, indicating that potential differences in inoculum potential among morphotypes at the start of the experiment did not cause any systematic competitive exclusion. Thus, future research should increasingly focus on the challenging topic of how different AMF (and their abundances) interact and how such interactions depend and feedback on community structure or other environmental factors.

In our experiments we used AMF morphotypes of different families, for which one may expect a higher degree of functional complementarity [7]. It is well documented, for instance, that AMF of the Gigasporacaea family tend to have higher extraradical hyphal lengths and lower or delayed intraradical root colonization than *Glomus* spp., which tend to produce more spores [7], [29], [43]. We assessed the spore formation of different morphotypes and our findings are consistent with *Glomus* spp. producing more spores than AMF of the Gigasporaceae. In Experiment 2, however, Gigasporaceae morphotypes represented a considerable proportion of the total AMF spore volume due to their relatively large spore sizes. Gigasporaceae AMF reportedly have a delayed root colonization compared to other AMF [43], possibly because their spores are the most important infective units (propagules) after disturbance; other AMF regrow from spores as well as other propagules such as dried colonized root or hyphal fragments [44]. Finally, in our experiments all AMF taxa persisted in AMF mixtures and no morphotype was consistently excluded, which is in line with a recent study that found the highest realized AMF species richness in phylogenetically overdispersed AMF communities [7]. In comparison, we used phylogenetically similarly dispersed (although species-poorer) AMF mixtures, which may explain why we did not detect such a strong phylogenetic signal.

In summary our study provides new insights into how functional complementarity of different AMF explains, at least in part, the enhanced AMF productivity or co-existence in diverse AMF assemblages. At the level of trophic groups, however, such functional differences seemingly “evened out” with increasing diversity: Overall both AMF and plant productivity were mainly determined by the diversity at the two trophic levels, with no interaction among them. We also found that whole-pot evapotranspiration rates in Experiment 1 were affected by AMF inoculation treatments. Similar effects in the field could potentially create localized soil moisture gradients that may also affect subsequent community assembly. Thus, our study also provides a basis for more eco-physiologically based studies, to test how AMF may impact the water use efficiency of vegetation or different host species.

In natural communities multiple mechanisms operate at different temporal and spatial scales to shape species distributions. Even though AMF have the potential to alter plant communities and whole ecosystem properties [16], [17], [45], little is known about key factors affecting their own growth, fitness, and dispersal in space and time [46], [47]. Variations of our approach should be applicable to more realistic microcosm or field experiments. A key challenge for future studies is to determine the relative importance of different types of interactions (*e.g.*, mycorrhizal symbioses, pathogens, competition, predation) for shaping succession dynamics, community structure and ecosystem functioning [45], [48]–[51]. While recent findings suggest that even climatic origin of AMF may affect plant growth [29], most current vegetation models do not incorporate biotic feedbacks, adding uncertainty to our understanding of how communities assemble in nature.
